# Cannabidiol (CBD) Supports the Honeybee Worker Organism by Activating the Antioxidant System

**DOI:** 10.3390/antiox12020279

**Published:** 2023-01-27

**Authors:** Patrycja Skowronek, Aneta Strachecka

**Affiliations:** Department of Invertebrate Ecophysiology and Experimental Biology, University of Life Sciences in Lublin, Doświadczalna 50a, 20-280 Lublin, Poland

**Keywords:** hemp extract, CBD oil, antioxidant enzymes, biochemistry, resistance, *Apis mellifera*, calcium

## Abstract

In the experiment, we tested the effect of 30% CBD oil on the activity of the antioxidant system (superoxide dismutase, catalase, glutathione peroxidase, glutathione), the level of total antioxidant capacity, and the concentrations of ions (calcium, magnesium, and phosphorus) in honeybee workers in the hive test. For this purpose, we prepared hives containing all stages of the development of honey bees and started the experiment by adding 200 marked, one-day old bees to each colony (intended for hemolymph collection). In the test, we created three groups (two colonies per group): (1) Experimental with CBD oil mixed with sugar syrup (CSy); (2) experimental with CBD oil on textile strips (CSt); and (3) control with pure sugar syrup only (C). Every week, we collected hemolymph from the marked bees. In the experiment, all antioxidant enzyme activities were higher for the experimental groups CSy and CSt compared to group C. The highest concentrations/levels were obtained for the CSy group. Concentrations of calcium, magnesium, and phosphorus ions were also higher for the experimental groups compared to the C group (the highest concentration for the CSy group). We conclude that CBD oil positively contributes to stimulating the antioxidant system of honeybees.

## 1. Introduction

Nowadays, the scientific world has been dealing with the problems of the decrease in the biodiversity of flora and fauna due to global environmental changes. Disruptions in the populations of living organisms cause wide-ranging changes that will affect all organisms on this planet. Among the frequently mentioned organisms, the populations of which are shrinking, are insects. As the most diverse group of organisms, it plays an important role in agriculture, from the decomposition of organic matter to pollination [1,2].

The beneficial insects that suffer from these negative effects are bees. As pollinators, bees are responsible for regulating the biodiversity of the natural environment. Their work includes the pollination of entomophilic plants, which include about 70% of plants that are part of the human diet and livestock and pets. In recent years, with the increase in the ecological crisis and problems with food production, it is extremely important to maintain the health and good condition of the populations of these insects. Due to the changing environment as a result of climate change, anthropopressure, and chemization, the natural resistance of bees decreases, and thus they are more susceptible to infections with parasites and pathogens (i.e., nosematosis, varroosis). All of these factors, acting together or separately, make a significant contribution to reducing the work efficiency and shortening the life of pollinators. The dying of colonies also causes far-reaching economic losses in the beekeeping, agricultural, and food industries [3,4,5,6,7,8]. In response to this crisis caused by the general weakening of the population of bee colonies, various supplements have been tested to both stimulate the health potential and meet a number of requirements (i.e., non-toxicity). Due to the keeping of honey bees in breeding farms from which bee products are obtained, the substances administered to the hive should be safe in the event that residues of these stimulants pass into food products (e.g., honey) [9,10].

Therefore, the most commonly used substances come from natural sources (i.e., plants with documented properties and classified as safe for human and animal health). Thus far, the tested substances of this type include resveratrol (active substance from grapes), piperine (from pepper), curcumin (turmeric), caffeine (present in coffee beans, cocoa beans and tea), vitamin C (parsley, lemon), and mixtures of pulses (soybean, corn, pea) and meals. Popularly used supplements also include the recently popular silages rich in Lactobacillus, phytochemicals stimulating the diversity of the intestinal microflora of bees (also Lactobacillus), coenzyme Q10 (pharmaceutical raw material), propolis (propolis tincture), spirulina, yeast, powdered skim milk, and pollen [11,12,13,14,15,16,17,18,19,20,21,22]. Most of the mentioned biostimulants prolonged the life of bees. Some of the tested substances had a proven positive effect on the stimulation of the immune system by increasing the activity of the proteolytic system, antioxidant enzyme like: SOD (superoxide dismutase), GpX (peroxidase), CAT (catalase), GST (glutathione S-transferase), and the concentration of immune proteins (hemp extract, curcumin, coenzyme Q10, caffeine, piperine). The addition of coenzyme Q10 additionally increased the concentration of lipids and ions such as magnesium and calcium. Higher lipid levels were noted after feeding bees with spirulina. Caffeine, in addition, had a positive effect in the case of bees infected with Nosema spp. and the test reported higher concentrations of proteins, urea acid, triglycerides, cholesterol, glucose, calcium, creatinine, magnesium, and proteases. The greatest stimulating effect was observed in older bees. The activities of liver enzymes (transaminases) were also higher in the piperine-treated group. Piperine decreased the DNA methylation levels significantly in the older bees [12,15,16,20,22,23]. Natural substances used in recent years also include eucalyptus pollen from *Corymbia calophylla*. Mixtures with linoleic acid, oleic acid, soybean meal, and lupin meal were used. The life-prolonging effect has been reported with the exclusive use of pollen alone. Each addition to the pollen resulted in a shortened lifespan. Oleic acid showed the weakest effect on bees [24]. Despite the large number of tested substances, it has not been possible to find one effective agent that suits all the needs of modern beekeeping and the strict requirements for the safety of use.

We believe that a good supplementation can be the addition of hemp extract in the form of CBD oil to the diet of bees, which has many documented potentially health-promoting properties. In the latest research, more and more scientists are showing the positive effects of cannabis on animal organisms. According to Boldaji et al. (2022), cannabidiol contributed to better outcomes in myocardial rehabilitation after myocardial infarction in rat tissue tests [25]. The research carried out by Majewski et al. (2021), also conducted on rats, confirmed that cannabis had a positive effect on the body by changing the blood biochemical parameters (lowering cholesterol levels, reducing lipid peroxidation, increasing the sensitivity of ATP, and calcium ion-dependent potassium channels) [26]. Studies have also been carried out on the regeneration of other (skeletal) muscles for sports performance in humans. It was noted that the CBD consuming group showed little but significant differentiation in muscle recovery after resistance training [27]. In other animal studies, horses that consumed CBD showed lower reactivity compared to the control groups, which may contribute to their welfare in the future [28]. It has also shown a positive effect on the antioxidant system by improving superoxide dismutation in dogs. The addition of hemp oil to the diet of these dogs also improved the digestibility of nutrients [29].

Our previous studies have shown that CBD oil/hemp extracts added to the diet of bees prolonged the life of insects and contributed to a positive stimulation of the immune system by stimulating the activity of the proteolytic system, increasing the enzymatic concentrations (ALT, AST, ALP), and non-enzymatic biomarkers (e.g., glucose, cholesterol, triglycerides, calcium, and magnesium ions) tested from the hive test. Additionally, in previous studies, we showed that supplementation with hemp extract increased the activity of the antioxidant system in the cage experiment (all enzymes) and prolonged the life of bees in cage experiments. Therefore, we assumed that in the case of a beekeeping experiment, we will obtain results confirming the positive effect of CBD oil obtained in the case of previous publications using a hemp extract. In addition, the results obtained in this publication will complement the information on CBD supplementation from previous studies on the proteolytic system [10,23,30].

The antioxidant system is a very important element of the immunity of living organisms. It is responsible for scavenging free radicals, which can cause the body to age faster, create inflammatory processes, and mediate in creating diseases and cancers. The main defense consists of antioxidant enzymes: superoxide dismutase (SOD), catalase (CAT), glutathione peroxidase (GPx), and glutathione (GSH). All of these enzymes interact with each other to neutralize reactive oxygen species (ROS) by transforming it into hydrogen peroxide and molecular oxygen (SOD), then into molecular oxygen and water (CAT, GPx, GSH) [31,32].

The aim of this study was to evaluate the effect of a commercial hemp extract in the form of CBD oil on the activity of the antioxidant system (TAC—total antioxidant capacity, SOD, CAT, GPx, GSH) and the basic ion concentrations important in cellular processes (calcium, magnesium, and phosphorus) in honey bees (*Apis mellifera*).

## 2. Materials and Methods

The research was carried out with the use of bees and queens from colonies of similar strength and age (number of brood, mother’s age, number of bees, frame construction, amount of feed stock, varroosis free, Nosema free, etc.) that had not been subjected to prophylactic treatment against *Varroa destructor* (and thus obtained the natural activity of their immune systems without the healing agents affecting them). No spores of *Nosema* spp. were detected in the colonies.

### 2.1. Preparatory Activities

#### 2.1.1. Obtaining Queens for the Experiment 

Nine queens were prepared for the experiment (six queens for the colony test + three queens for day-old workers). All nine queens were sisters descended from one source mother (all mothers were inseminated). The methodology obtained mothers according to Skowronek et al. [23].

#### 2.1.2. Preparation of the Mating Hives

Four-frame hives were used for the experiment.

Fragments of various combs were collected from the source colony, in which we observed different stages of larvae development. Fragments were fitted to frames in the experimental hives. Additionally, worker bees of different ages (imago stages) were collected from the source colony as a complement to the colony, and 200 individuals were placed in each of the prepared hives. Each hive contained all development stages. The queen was put into each hive (six queens = six hives). We checked the queen abilities to lay eggs after 1 month and next we added 1 day old bees to the colonies, obtained from the three remaining queens—sisters [23,33].

### 2.2. Collection and Tagging of Day-Old Bees

Three queen-sisters were caged for 12 h in a queen excluder comb-cage containing one empty comb for egg laying. When the queens laid eggs, we released them to the source colonies. The combs with eggs were marked and placed in their native colonies. After 20 days, these combs with broods were placed in an incubator (35 °C) where the 1-day-old bees emerged. More details about the collection and marked one-day-old bees from three mother-sisters are described in Skowronek et al. [23].

One-day workers were placed in each of the six mating hives. In the experiment, the hives were divided into three groups: (1) The experimental group CSy, CBD in sugar syrup; (2) experimental group CSt, CBD on a cotton strip; and (3) the C control (supplemented with pure sugar syrup). Each group consisted of two mating hives [23,34].

### 2.3. Preparation and Administration of CBD Extract

Hemp extract in the form of 30% CBD oil (producer’ name: HempOil, 3 g of CBD in 10 mL of oil) was used for the experiment. The oil was obtained by CO_2_ extraction (producer information). Supplementation details for the experimental groups are as follows:

– CSy: ad libitum, on the second, fourth, and sixth days of the experiment, in a mixture with sugar syrup (1:1 water with sugar) and glycerin in the volume ratio of 0.01:0.5:0.5 (extract:distilled water:glycerin);
– CSt: mixture with water and glycerin in a volume ratio of 0.8:1.5:1.5 (extract:distilled water:glycerin), textile strips (2 × 10 cm) were evenly moistened with 10 mL of the mixture and placed in the hives, wetted with the mixture on the second, fourth, and sixth days of the experiment.

Details on the composition of supplementation according to Skowronek et al. [23].

### 2.4. Sampling of Bees

From six colonies, we collected 10 marked bees once per week (10 bees × 6 colonies). Workers were collected on the following days of the experiment: 2, 7, 14, 21, 28 (CSt, CSy, C), and 35 (CSy) [23].

### 2.5. Collection of Hemolymph

The hemolymph samples were collected from each worker according to the methodology of Łoś and Strachecka (2018) using capillary tubes (20 µL; end to end type; no anticoagulant; Medlab Products, Raszyn, Poland). The hemolymph capillaries of the individual bees were placed in separate 1.5 mL Eppendorf tubes with a 200 µL 0.6% NaCl solution (10 bees = 10 tubes). Then, the material was stored at −25 °C [35].

### 2.6. Biochemical Analyses

#### 2.6.1. Antioxidant System

The analyses were carried out using the commercial assay kit:

– TAC—OxiSelectTM Total Antioxidant Capacity Assay Kit (Cell BioLabs, Inc., Upper Heyford, UK, no. STA-360);
– SOD Assay Kit (Sigma Aldrich, Schnelldorf, Germany, no. 1916-1KT-F);
– CAT Assay Kit (Sigma Aldrich, Schnelldorf, Germany no. CAT100-1KT);
– GPx—Glutathione peroxidase Assay Kit (Sigma Aldrich, Schnelldorf, Germany, no. MAK437);
– GSH—EnzyChromTM GSH/GSSG Assay Kit (Bio Assay Systems, Hayward, CA, USA, no. EGTT-100).

All antioxidant enzyme activities were calculated per 1 mg of protein. 

The original protocols owned by the companies are available in an electronic version on the manufacturers’ website.

#### 2.6.2. Ions Concentration

The ion concentrations were analyzed using commercial one-component reagents:

– Calcium (Alpha Diagnostics, arsenazo III method, reagent composition: TRIS buffer pH 8.5; arsenazo III, 8-hydroxy-quinoline-5-sulfonic acid, inactive stabilizers and detergents 630–670 nm);
– Magnesium (Alpha Diagnostics Magnesium Xylidyl Blue, reagent composition: trioglycolic acid, DMSO, Xylidyl Blue, measurement at 550 nm);
– Phosphorus (Alpha Diagnostics, direct method with phosphomolybdate, reagent composition: sulfuric acid, ammonium molybdate, measurement at 340 nm).

### 2.7. Statistical Analysis

The results were analyzed using Statistica formulas version 13.3 (2017) for Windows (StatSoft Inc., Tusla, OK, USA). The mixed-model two-way ANOVA followed by Tukey HSD post hoc tests (*p* = 0.05) was used to compare the results for each antioxidant enzyme (SOD, GST, CAT, GPx, and TAC) and the ions (calcium, magnesium, phosphorus) of the honey bee workers, depending on the method of administration (strip—CSt and syringe—CSy) and the day (1st, 7th, 14th, 21st, 28th, 35th) of supplementation with CBD oil.

## 3. Results

### 3.1. Antioxidant Enzymes

**Figure 1:**
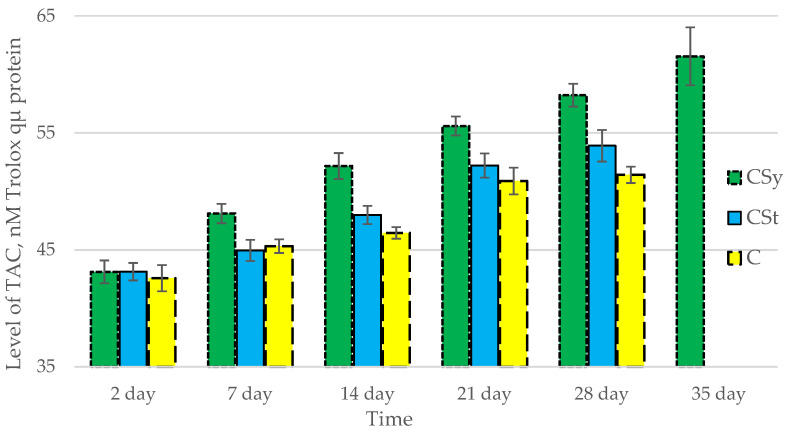
Total Antioxidant Capacity (TAC) levels in the 2-, 7-, 14-, 21-, 28-, 35-day-old honey bees’ hemolymph after two methods of supplementation with hemp extract: C—control (pure sugar syrup), Csy—hemp extract in syrup, Cst—hemp extract on strips. (Two-Way ANOVA: supplementation method: F_(2,146)_ = 100,26, *p* = 0.0000; days of supplementation: F_(4,138)_ = 506,23, *p* = 0.0000; supplementation method × days of supplementation F_(8,138)_ = 13,046, *p* = 0.0000.

**Figure 2:**
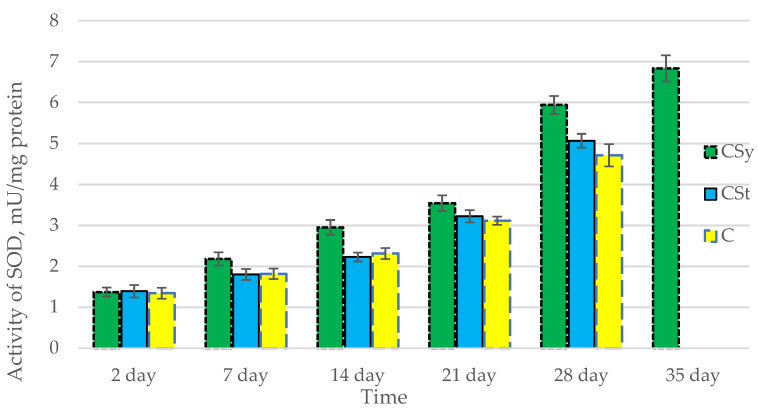
Activities of Superoxide Dismutase (SOD) in the 2-, 7-, 14-, 21-, 28-, 35-day-old honey bees’ hemolymph after two methods of supplementation with hemp extract: C—control (pure sugar syrup), Csy—hemp extract in syrup, Cst—hemp extract on strips. (Two-Way ANOVA: supplementation method: F_(2,146)_ = 66,223, *p* = 0.0000; days of supplementation: F_(4,138)_ = 1888,5, *p* = 0.0000; supplementation method × days of supplementation F_(8,138)_ = 16,781, *p* = 0.0000.

**Figure 3:**
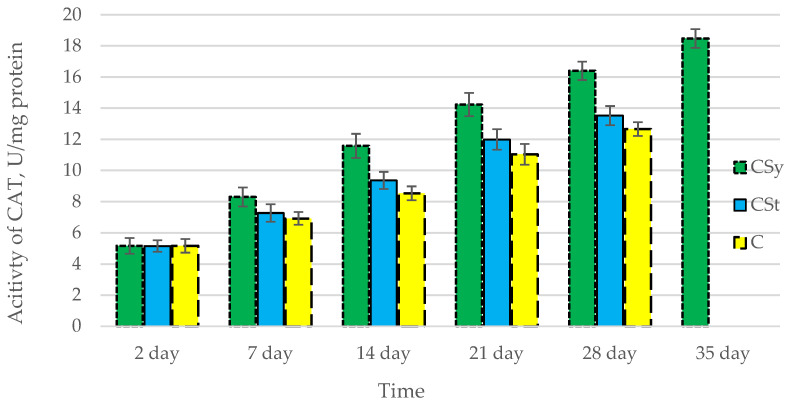
Activities of Catalase (CAT) in the 2-, 7-, 14-, 21-, 28-, 35-day-old honey bees’ hemolymph after two methods of supplementation with hemp extract: C—control (pure sugar syrup), Csy—hemp extract in syrup, Cst—hemp extract on strips. (Two-Way ANOVA: supplementation method: F_(2,146)_ = 96,388, *p* = 0.0000; days of supplementation: F_(4,138)_ = 1070,6, *p* = 0.0000; supplementation method × days of supplementation F_(8,138)_ = 18,549, *p* = 0.0000.

**Figure 4:**
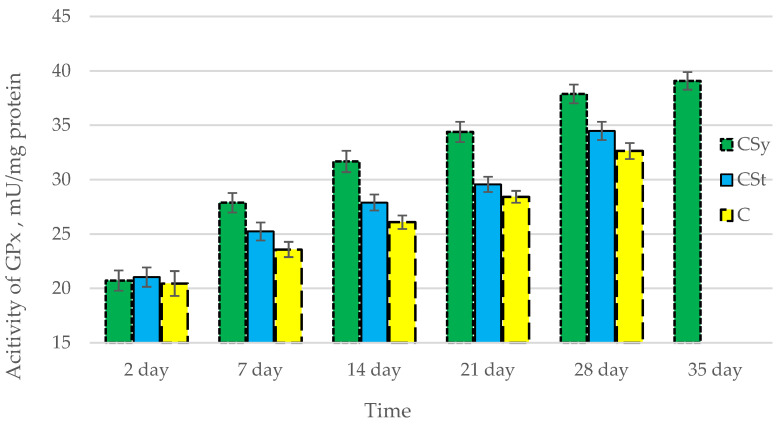
Activities of Glutathione Peroxidase (GPx) in the 2-, 7-, 14-, 21-, 28-, 35-day-old honey bees’ hemolymph after two methods of supplementation with hemp extract: C—control (pure sugar syrup), Csy—hemp extract in syrup, Cst—hemp extract on strips. (Two-Way ANOVA: supplementation method: F_(2,146)_ = 145,31, *p* = 0.0000; days of supplementation: F_(4,138)_ = 1093,6, *p* = 0.0000; supplementation method × days of supplementation F_(8,138)_ = 22,266, *p* = 0.0000.

**Figure 5:**
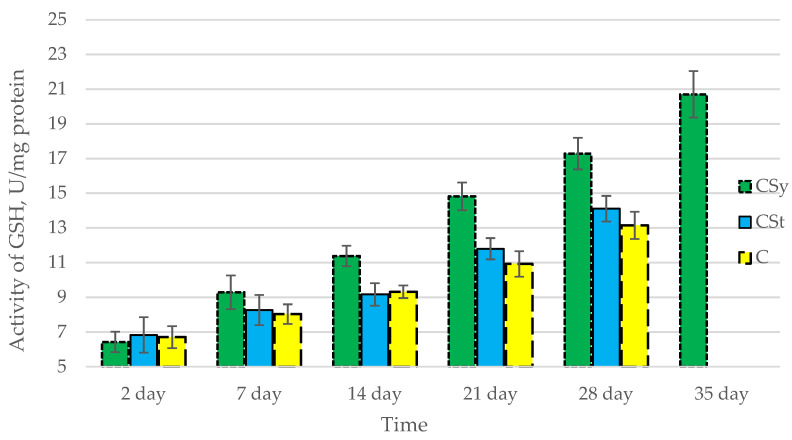
Activities of Glutathione (GSH) in the 2-, 7-, 14-, 21-, 28-, 35-day-old honey bees’ hemolymph after two methods of supplementation with hemp extract: C—control (pure sugar syrup), Csy—hemp extract in syrup, Cst—hemp extract on strips. (Two-Way ANOVA: supplementation method: F_(2,146)_ = 56,662, *p* = 0.0000; days of supplementation: F_(4,138)_ = 435,40, *p* = 0.0000; supplementation method × days of supplementation F_(8,138)_ = 14,742, *p* = 0.0000.

The total antioxidant capacity and all antioxidant enzyme activities (SOD, CAT, GPx, GSH) achieved higher values for the groups in which bees were exposed to CBD oil (CSy, CSt) compared to the control group (C). The highest activities were recorded for bees that directly consumed sugar syrup with CBD oil (CSy). The values of the activity in each parameter increased with the age of the bees (Figure 1, Figure 2, Figure 3, Figure 4 and Figure 5, Table 1).

### 3.2. Ion Concentrations

**Figure 6:**
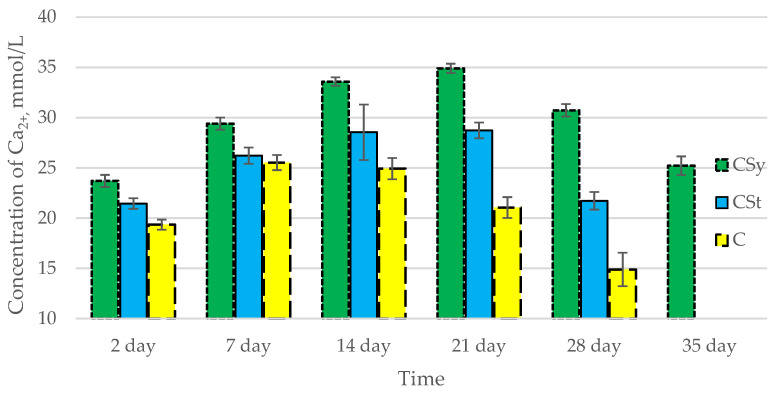
Calcium concentrations in the 2-, 7-, 14-, 21-, 28-, 35-day-old honey bees’ hemolymph after two methods of supplementation with hemp extract: C—control (pure sugar syrup), Csy—hemp extract in syrup, Cst—hemp extract on strips. (Two-Way ANOVA: supple-mentation method: F_(2,146)_ = 213,61, *p* = 0.0000; days of supplementation: F_(4,138)_ = 310,89, *p* = 0.0000; supplementation method × days of supplementation F_(8,138)_ = 66,793, *p* = 0.0000.

**Figure 7:**
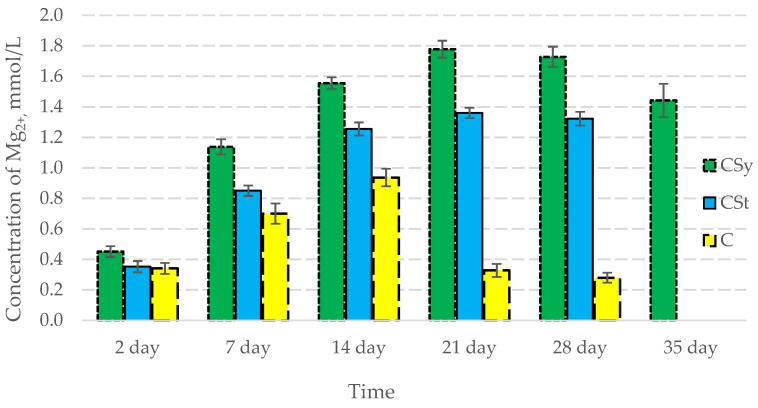
Magnesium concentrations in the 2-, 7-, 14-, 21-, 28-, 35-day-old honey bees’ hemolymph after two methods of supplementation with hemp extract: C—control (pure sugar syrup), Csy—hemp extract in syrup, Cst—hemp extract on strips. (Two-Way ANOVA: supplementation method: F_(2,146)_ = 145,02, *p* = 0.0000; days of supplementation: F_(4,138)_ = 1312,0, *p* = 0.0000; supplementation method × days of supplementation F_(8,138)_ = 379,63, *p* = 0.0000.

**Figure 8:**
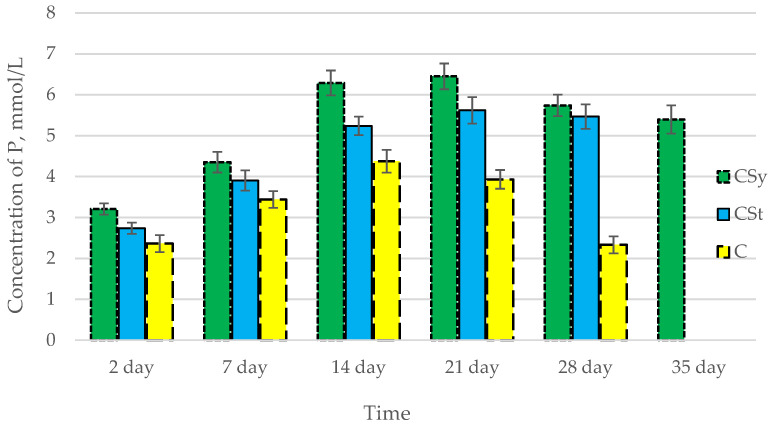
Phosphorus concentrations in the 2-, 7-, 14-, 21-, 28-, 35-day-old honey bees’ hemolymph after two methods of supplementation with hemp extract: C—control (pure sugar syrup), Csy—hemp extract in syrup, Cst—hemp extract on strips. (Two-Way ANOVA: supplementation method: F_(2,146)_ = 166,54, *p* = 0.0000; days of supplementation: F_(4,138)_ = 530,24, *p* = 0.0000; supplementation method × days of supplementation F_(8,138)_ = 65,556, *p* = 0.0000.

In all of the tested ions, we observed higher concentrations of calcium, magnesium, and phosphorus ions for the experimental groups with CBD oil (CSy, CSt) compared to the control group (C). The highest concentrations were achieved by bees consuming the addition of CBD oil in the sugar syrup. For all groups, the concentrations of ions increased until day 21, and on day 28, we recorded a downward trend for the concentrations of all of the tested ions (Figure 6, Figure 7 and Figure 8, Table 2).

**Figure:**
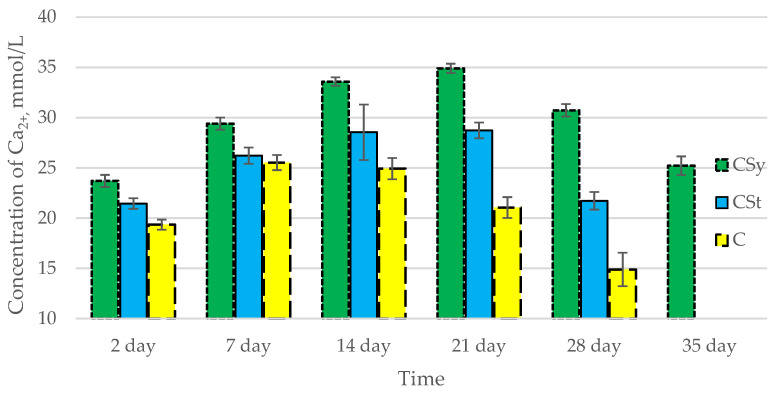


## 4. Discussion

In our study, it turned out that all of the activities of the antioxidant enzymes tested by us were higher for the experimental groups with CBD oil, in particular, the best stimulation effect was obtained for the group consuming the addition of this oil. The results obtained in this article coincide with our previous publications on the antioxidant system in a cage experiment and the proteolytic system in apiary conditions [10,23,30]. In addition, by examining the concentrations of ions, in particular calcium ions, it turns out that the mechanism of CBD action assumed by us in bees is correct. The increase in the concentrations of calcium ions in the experiment suggests that it may have an effect on the ion-dependent immune enzymes involved in the body’s defense (i.e., specific calcium phospholipases such as cytosolic phospholipase (A2), dependent on cPLA2-Ca^2+^). Phospholipase A2 (PLA2) is of key importance in the synthesis of eicosanoids, prostaglandins, and the production of lipoxygenase products (by the hydrolysis of polyunsaturated fatty acid, linoleic acid, and conversion to arachidonic acid), which can exert positive and negative effects on immunity depending on the age of insects (prostaglandin can cause inflammation in older individuals, but is a positive factor in the development of young individuals). PLA2 is activated in insects immediately after infection is detected, thanks to which the body quickly reacts to the threat of eicosanoid synthesis [30,36,37,38]. Eicosanoids are responsible for many mechanisms of cellular resistance to infection, invasions (phagocytosis and mediating the production of reactive oxygen species used to kill microbes) and wounding (melanization), influencing the production of nodules, signals of hemocyte migration. It is also responsible for the signal to release profenoloxidase (in Lepidoptera) [37]. Higher calcium levels, better phospholipase A2 performance, and eicosanoid synthesis may also be related to the antioxidant system in this study. According to the research by Büyükgüzel et al. (2010) and Büyükgüzel et al. (2017), the inhibition of eicosanoid synthesis increases oxidative stress in the insect organism and increases lipid peroxidation. The authors suggest that eicosanoids may be an intermediate in the action and activities of the antioxidant system in invertebrates by also mediating the production of reactive oxygen species during the phagocytosis process [38,39,40,41]. Thus, in a sense, the synthesis of eicosanoids and their activities in the body may contribute to the stimulation of antioxidant enzymes. In addition, the calcium and potassium ions (we noticed an increase in concentration) suggest the correctness of the assumption that CBD may affect the permeability of ion channels (sodium, potassium, and calcium) and their integrity (also suggested by the increase in magnesium concentration) in the cell membrane. Higher ion concentrations may contribute to higher activities and the production of antioxidants (e.g., catalase), the formation of which depends on the amount of calcium, which affects the level of H_2_O_2_ (catalase synthesis) [42,43,44]. In the case of the production of ROS by eicosanoids and the dependence of the production of antioxidant enzymes on its level, it is also possible that by giving CBD oil, it caused a temporary increase in ROS. Thanks to this, we stimulated an increase in the activity of the antioxidant system (stimulation of higher mRNA expression in the early stages of life), as a result of which we obtained higher concentrations in the later stages of the life of bees (higher level of enzymes in early bee life stages caused better resistance for ROS in later stages of bee life). We believe that the increased immunity at an early age increased the immunity of the bees collected, which upon leaving the hive were exposed to oxidative stress in the natural environment [44,45,46]. On the other hand, studies by Zhang et al. (2022) suggest that CBD in the *Caenorhabditis elegans* insect test produced a positive effect on the body without the additional overexpression of antioxidant genes. Regardless of the process, these studies suggest a significant effect of CBD on the possibility of inhibiting the progress of Alzheimer’s disease and that CBD substances themselves (thanks to the presence of phenolic groups) are excellent at scavenging free radicals in vitro and in vivo [47]. As with previous articles, we observed the same trends between the bees that had been exposed to CBD on the strip and the bees that consumed the CBD supplement. We maintain the opinion that the better effect of stimulating the immune system of bees in the CSy group is due to the direct entry of supplementation into the digestive system of insects, thanks to which the active substances have a chance to act faster. In the case of the CSt groups, we suggest that the lower effect is due to the longer time that CBD takes to enter the body, probably through tropholaxy and hygienic behavior in bees (cleaning). The stimulation of the antioxidant system is a positive effect, which can be confirmed by other publications in the field of bee supplementation conducted by Strachecka et al. In studies with the use of other strong antioxidants (i.e., coenzyme Q10 and curcumin and other biostimulants like caffeine), the same increase in the activities of selected antioxidant enzymes was noted. The positive effect in the cases of the above-mentioned publications and in our article was confirmed by the extension of the life span of bees. In the case of all supplementation, an increase in the concentrations of calcium and magnesium ions were also noted [15,16,20].

## 5. Conclusions

After a series of studies in cages and in colony conditions, we found that supplementation with CBD will potentially support the immune system of honeybees through stimulating the antioxidant system (protection against oxidative stress affecting cells and their biochemistry). Depending on the need, the effects can be obtained regardless of the method of administration, but for the best results, we suggest using CBD in nutritional supplements (direct, faster action). In addition, research confirms that the active substance CBD may be responsible for the positive effect of the hemp extract.

## Figures and Tables

**Table 1:** Standard error for activities for antioxidant enzymes (for all groups from 2–35 days during the experiment) in hemolymph samples after two methods of supplementation with the hemp extract: C—control (pure sugar syrup), Csy—hemp extract in syr-up, Cst—hemp extract on strips, according to two-way ANOVA.

Groups	Day of Supplementation	se±				
		TAC	SOD	CAT	GPx	GSH
CSt	2 days	0.348525	0.054445	0.182303	0.264725	0.250834
	7 days	0.348525	0.054445	0.182303	0.264725	0.250834
	14 days	0.348525	0.054445	0.182303	0.264725	0.250834
	21 days	0.348525	0.054445	0.182303	0.264725	0.250834
	28 days	0.367377	0.05739	0.192164	0.279045	0.264402
	35 days	-	-	-	-	-
CSy	2 days	0.348525	0.054445	0.182303	0.264725	0.250834
	7 days	0.348525	0.054445	0.182303	0.264725	0.250834
	14 days	0.348525	0.054445	0.182303	0.264725	0.250834
	21 days	0.348525	0.054445	0.182303	0.264725	0.250834
	28 days	0.348525	0.054445	0.182303	0.264725	0.250834
	35 days	0.348525	0.054445	0.182303	0.264725	0.250834
C	2 days	0.348525	0.054445	0.182303	0.264725	0.250834
	7 days	0.348525	0.054445	0.182303	0.264725	0.250834
	14 days	0.348525	0.054445	0.182303	0.264725	0.250834
	21 days	0.348525	0.054445	0.182303	0.264725	0.250834
	28 days	0.492888	0.076997	0.257815	0.374378	0.354732
	35 days	-	-	-	-	-

**Table 2:** Standard error for the calcium, magnesium, and phosphorus ions (for all groups from 2–35 days during the experiment) in hemolymph samples after two methods of supplementation with the hemp extract: C—control (pure sugar syrup), Csy—hemp extract in syrup, Cst—hemp extract on strips, according to two-way ANOVA.

Groups	Day of Supplementation	se±		
		Ca^2+^	Mg^2+^	P
C	2 days	0.336983	0.016605	0.080677
	7 days	0.336983	0.016605	0.080677
	14 days	0.336983	0.016605	0.080677
	21 days	0.336983	0.016605	0.080677
	28 days	0.336983	0.016605	0.080677
	35 days	-	-	-
CSt	2 days	0.336983	0.016605	0.080677
	7 days	0.336983	0.016605	0.080677
	14 days	0.336983	0.016605	0.080677
	21 days	0.336983	0.016605	0.080677
	28 days	0.355211	0.017503	0.085041
	35 days	-	-	-
CSy	2 days	0.336983	0.016605	0.080677
	7 days	0.336983	0.016605	0.080677
	14 days	0.336983	0.016605	0.080677
	21 days	0.336983	0.016605	0.080677
	28 days	0.336983	0.016605	0.080677
	35 days	0.336983	0.016605	0.080677

## Data Availability

The datasets and materials for the study results that were used, analyzed, and presented in this manuscript are not publicly available, but are available at the University of Life Sciences in Lublin. At the justified request of the interested party, they may be made available by the respective author.
